# Nanotechnologies in Food Science: Applications, Recent Trends, and Future Perspectives

**DOI:** 10.1007/s40820-020-0383-9

**Published:** 2020-02-04

**Authors:** Shivraj Hariram Nile, Venkidasamy Baskar, Dhivya Selvaraj, Arti Nile, Jianbo Xiao, Guoyin Kai

**Affiliations:** 1 Laboratory of Medicinal Plant Biotechnology, College of Pharmacy, Zhejiang Chinese Medical University, Hangzhou, 310053 Zhejiang People’s Republic of China; 2 Plant Genetic Engineering Laboratory, Department of Biotechnology, Bharathiar University, Coimbatore, Tamil Nadu India; 3 Department of Bioresources and Food Science, Sanghuh College of Life Sciences, Konkuk University, Seoul, 05029 Republic of Korea; 4 Institute of Chinese Medical Sciences, State Key Laboratory of Quality Control in Chinese Medicine, University of Macau, Macau, Macau SAR People’s Republic of China

**Keywords:** Nanomaterials, Functional food, Food processing, Nanodelivery, Bioavailability

## Abstract

Nanotechnology is a key advanced technology enabling contribution, development, and sustainable impact on food, medicine, and agriculture sectors. Nanomaterials have potential to lead qualitative and quantitative production of healthier, safer, and high-quality functional foods which are perishable or semi-perishable in nature. Nanotechnologies are superior than conventional food processing technologies with increased shelf life of food products, preventing contamination, and production of enhanced food quality. This comprehensive review on nanotechnologies for functional food development describes the current trends and future perspectives of advanced nanomaterials in food sector considering processing, packaging, security, and storage. Applications of nanotechnologies enhance the food bioavailability, taste, texture, and consistency, achieved through modification of particle size, possible cluster formation, and surface charge of food nanomaterials. In addition, the nanodelivery-mediated nutraceuticals, synergistic action of nanomaterials in food protection, and the application of nanosensors in smart food packaging for monitoring the quality of the stored foods and the common methods employed for assessing the impact of nanomaterials in biological systems are also discussed.

## Highlights

- Different nanotechnologies and nanomaterials with their efficient applications in functional food development are summarized.
- Nanotechnologies boosted the food, medicine, and biotechnology sector through enhanced food bioavailability, food processing, packaging, and preservation are also reviewed.
- This comprehensive review on nanotechnologies in food science describes the recent trend and future perspectives for future functional nanofood research and development.

## Graphical abstract

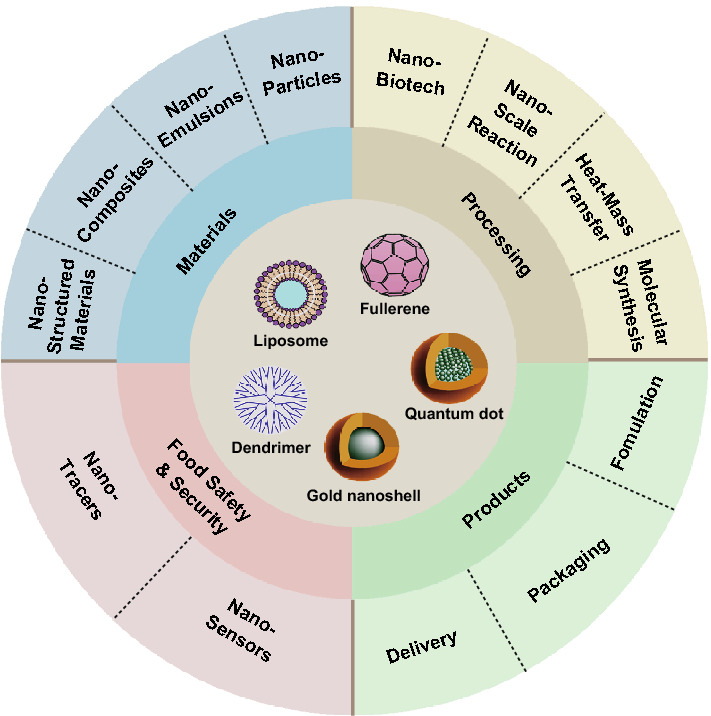

## Introduction

**Fig. 1:**
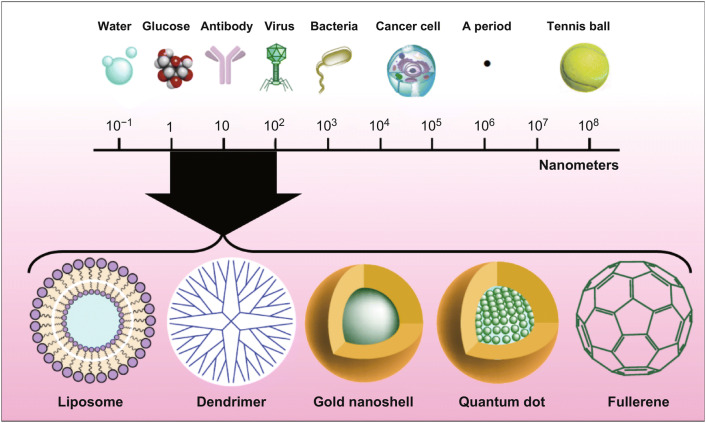
Different sized nanoparticles used in nanotechnologies of food science. This figure was modified and adopted from Ref. [255] with permission

Nanotechnology is the technology applied in the manipulation of nanomaterials for several purposes, which plays a crucial role in the food and agriculture sectors, contributes to crop improvement, enhances the food quality and safety, and promotes human health through novel and innovative approaches [1]. Owing to the unique physical, chemical, and biological properties with large surface–volume ratio as well as the altered solubility and toxicity when compared with their macroscale counterparts, engineered nanometer-sized particles have gained more attention in medicine, agro-food sectors, sewage water treatments, and other industries [2, 3]. Silver (Ag), gold (Au), zinc oxide (ZnO), titanium dioxide (TiO_2_), and carbon nanoparticles are manufactured as much as tenfold that of other nanomaterials in amount due to their potential antimicrobial characteristics, being used in air filters, food storage containers, deodorants, bandages, toothpastes, paints, and other home appliances [3, 4]. Besides, the potent antibiotic activity of nanosized copper oxides (nCuO) has resulted in the wide application in commercial nano-biocide products [5]. Nanomaterials are tiny particles ranging from 1 to 100 nm in size, insoluble or bio-persistent in nature, synthesized through various routes, and used in numerous fields including medicine, electronics, agriculture, and food industries [6]. Different sized nanoparticles are used in nanotechnologies of food science for potential production and processing of healthier, safer, and high-quality foods (Fig. 1).

**Fig. 2:**
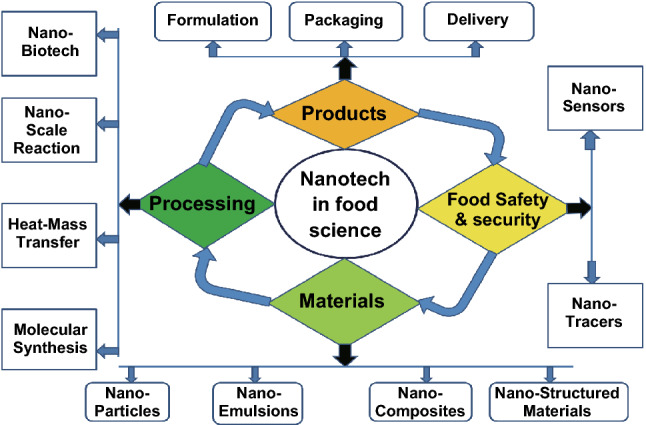
Nanotechnology in food science and technology

Food wastage leads to major losses in the food industry. Food and Agriculture Organization of the United Nations reported that more than 1.3 billion metric tons of consumable food is lost or wasted every year throughout the supply chain, mainly because of inferior post-harvest techniques, storage, transport facilities, and market and consumer wastage of food [7]. Apart from enhancing the food production rate, it is mandatory to tackle food wastage for the purpose to solve the food crisis caused by the emerging population and environmental issues. The major cause for food wastage is microbial contamination and food spoilage that reduces the food quality and affects food security, decreases shelf life of food products, and increases the risks of food-borne diseases [8]. In food industry, nanotechnology is applied for all practices: food production, processing, storage, and distribution (Fig. 2). It provides enhanced security by using nanosensors to detect any pathogen or containments in food. Nanotechnology-enhanced food packaging offers an improvement over conventional packaging that uses plastic barriers, and at the same time its functional components such as antimicrobial activities provide increased shelf life to the food products. It is also involved in the detection of food toxins, flavor production, and color formation [9]. Nanotechnology-based smart and intelligent systems provide localization, sensing, reporting, and remote control of food items with improved efficiency and security. Furthermore, nano-based delivery systems improve the nutraceutical values of the food components. Apart from their roles in food industry, nanomaterials also promote plant growth. For instance, TiO_2_ was shown to enhance the growth of many plants, gold nanoparticles increased the yield of seeds in *Arabidopsis*, and cellulose nanocrystals boost seed germination owing to their high water uptake potential [10].

The effective antimicrobial nature of biologically synthesized metallic nanoparticles not only controls the plant diseases, but also decreases environmental pollution. Nanomaterials, like carbon nanotubes, act as potential antimicrobial agents. Aggregates of carbon nanotubes caused cellular damage or death of *E. coli* by puncturing the cell when in direct contact with the bacterium [11]. The implementation of nano-biosensors in the detection of carcinogenic pathogens to prepare high-quality and contamination-free food has been widely reported. In this review, the potential utilization and applications of nanotechnology with different nanomaterials in food sector considering processing, preservation, storage, and security in terms of enhanced shelf life and quality are discussed. The potential applications of nanotechnology in nutraceuticals, the diagnosis of food pathogens as well as the possible harmful effects of nanotechnology in human and animal health are reviewed.

## Nanotechnology in Food Processing

Nanofood refers to the food generated by using nanotechnology in processing, production, security, and packaging of food. Nanotechnology has immense potential in the post-harvest food processing. It enhances food bioavailability, taste, texture, and consistency, or conceals the unpleasant taste or odor, and modifies the particle size, size distribution, possible cluster formation, and surface charge [12]. Edible nano-coatings (~ 5 nm thin coatings) can be used in meat, fruits, vegetables, cheese, fast food, bakery goods, and confectionery products, in which they serve as gas and moisture barriers. In addition, they provide flavor, color, enzymes, antioxidants, anti-browning compounds, and a prolonged shelf life to the manufactured products. Various bakery goods, coated with edible antibacterial nano-coatings, are available [13, 14]. Nanofilters have been used to remove color from beetroot juice while retaining the flavor and the red wine, and to remove lactose from milk so that it can be substituted with other sugars, making the milk suitable for lactose-intolerant patients. Nanoscale filters help in the elimination of bacterial species from milk or water without boiling. Nanomaterials used for developing nanosieves can be used for the filtration of milk and beer [9, 14].

Nanotechnology is used in the production of healthier food containing low fat, sugar, and salt to avoid food-borne diseases. It was reported that silicon dioxide (SiO_2_) and TiO_2_ were allowed as food additives in bulk quantities (E551 and E171, respectively) [15]. The shelf life of tomato has been increased by the bionanoencapsulated quercetin (biodegradable poly-D,L-lactide), and this approach should be extended to increase the shelf life of other vegetables and fruits [16]. Nanogreen tea, Neosino capsules (dietary supplements), Canola active oil, Aquanova (micelle to enhance the solubility of vitamins (A, C, D, E, and K), beta-carotene, and omega fatty acids), Nutralease (fortifying nanocarriers to carry nutraceuticals and drugs) are the common commercialized nanotechnology-based products in the market. Similarly, fortified fruit juices, oat nutritional drinks, nanoteas, nanocapsules containing tuna fish oil in breads, and nanoceuticals slim shakes are few commercially available nano-processed foods in the market which are widely sold in the USA, Australia, China, and Japan [17, 18]. Nanotechnology in production of commercial nanofood products and their applications in various food science technologies are shown in Table 1.

**Table 1:** Commercial nanofood products and their applications

Product name	Type of product	Manufacturer	Nanomaterial	Applications	References
Nutra Leaseanola Active Oil	Food and beverage	Shemen, Haifa, Israel	Nanosized self-assembled liquid structures (NSSL)	Inhibits transportation of cholesterol from the digestive system into the bloodstream	[21]
Nanotea	Beverage	Shenzhen Become Industry Trading Co. Guangdong, China	Nanoselenium	Good supplement of selenium	[173]
Fortified Fruit Juice	Health drink	High Vive. com, USA	Micelles 5–100 nm in diameter	Increased Lycopene	[173]
Nanoceuticals Slim Shake	Health drink	RBC Lifesciences, Irving, USA	Conversion of vanilla or chocolate into nanoscale	Low-calorie diet	[174]
NanoSlim beverage	Food and beverage	NanoSlim	Liquid suspended nanoparticle	Low-calorie diet	[174]
Oat Nutritional Drink	Food and beverage	Toddler Health, Los Angeles, USA	–	Contains exactly 33% of all the macro- and micronutrients	[175]
Tip Top bread	Food	George Weston Foods, Enfield, Australia	Nanosized self-assembled liquid structures	Nanocapsules of omega-3 fatty acids	[173]
Nano B-12 Vitamin Spray	Food supplements	Nanotech, LLC (USA)	Nanodroplets	Efficiency enhancement	[176]
Kimchi	Korean fermented cabbage dish	Korea	Nanometric *Lactobacillus plantarum*	Effective substituent for live probiotics and be useful as a functional ingredient with the anticolitic	[177]
Neosino	Health supplement	Germany	Silicon	Health and fitness	[176]
Aquanova	Food supplements	Germany	Nanomicelles	Improve the solubility of vitamins, β-carotenes, omega fatty acids	[178]
Oat Chocolate and Oat Vanilla Nutritional Drink	Beverage	Oat Chocolate and Oat Vanilla Nutritional Drink	300 nm of iron particles	Increases reactivity and bioavailability	[179]
Aquasol preservative	Food additive	Aquanova	Nanoscale micelle	Increases absorption and effectiveness of nutritional additives and preservatives	[178]
Omega-3	Food additive	Bioral™	Nanocochleates as small as 50 nm	Effective addition of omega-3 fatty acids	[180]
LycoVit	Food additive	BASF	< 200 nm synthetic lycopene	Potent antioxidant and used in soft drinks	[181]
Nano silver cutting board	Food contact material	A-Do Global	Nanoparticles of silver	Potent antibacterial	[181]
Antibacterial kitchenware	Food contact material	Nano Care Technology/NCT	Nanoparticles of silver	Increased antibacterial properties	[181]
Fresher Longer TM Miracle	Food storage	Sharper Image^®^, USA	25 nm of silver nanoparticles	Antimicrobial protection	[173]
Fresher Longer TM	Food storage	Sharper Image^®^, USA	Plastic	Longevity of food products	[173]
Nano Silver Food Containers	Food storage	A-DO Global, Korea	Silver	Storage	[173]
Nano Silver Baby Milk Bottle	Health benefits for toddler	Baby Dream^®^ Co. Ltd. (South Korea).	Nanosilver	Storage	[173]
Food storage containers	Food storage	BlueMoonGoods, LLC, USA	Silver	Food storage	[173]
Large Kitchen Appliances	Food storage	Daewoo^®^ Refrigerator, Korea	Nanosilver	Strong disinfection and storage power	[173]
Nano-silver Salad Bowl	Food storage	Changmin Chemicals, Korea	Silver	Storage	[182]
Nano Storage Box	Food storage	BlueMoonGoods™, USA	Silver	Food storage	[173]
Novasol	Sustain beverage	Aquanova^®^, Germany	Nanomicelle	Introduce antioxidant into food and beverage products	[173]
Nanoceuticals™	Nanosized powders	RBC Life Sciences^®^ Inc. (USA)	Nanocolloidal silicate mineral and Hydracel^®^	Neutralize free radicals, lower the surface tension of drinking water, and increase solvent properties	[173]
Nutri-NanoTM CoQ-10	–	Solgar (USA)	(~ 30 nm size)	Increased absorption fat	[183]
C.L.E.A.N. Products	–	SportMedix, Inc. (USA)	Nanostructured bioregulators	Normal functioning of organs and tissues	[173]
LifePak^®^ Nano	–	Pharmanex^®^ (USA)	–	Increases bioavailability	[173]
NanoCluster™	Spirulina nanoclusters	RBC Life Sciences^®^ Inc. (USA)	Nanoclusters of Artichoke, spirulina, and slim shake chocolate that contain cocoa nanoclusters	Enhances flavor	[173]
Nanocochleate nutrient	–	BioDelivery Sciences International’s BioralTM	Phosphatidylserine-based carrier system (~ 50 nm) derived from soya bean	Delivery system for micronutrients and antioxidants	[173]
Lypo-Spheric Vitamin C	Supplements	LivOn Labs, USA	Liposomal nanospheres	Health application	[173]
Daily Vitamin Boost	Fortified Jambu Juice	Hawaii, USA	Silver nanoparticle	Rich in 22 essential vitamins and minerals	[173]
SoluE	Vitamin E	Aquanova	–	Protects stomach from acidic environment	[173]
SoluC	Vitamin E	Aquanova	–	Protects stomach from acidic environment	[173]
Megace^®^ ES	Nanocrystal dispersion with micronized particles	Par Pharmaceutical, Inc., Bristol-Myers Squibb company, New York, USA	–	Appetite stimulant in case of cachexia	[173]
OilFresh™	Nanoceramic product	US-based Oilfresh Corporation	–	Suppresses oil breakdown	[184]
Bioral	Nanocochleate	BioDelivery Sciences International	Calcium ions in GRAS phosphatidylserine from soya bean	A protective delivery system for micronutrients and antioxidants against enzymatic degradation	[35]
ASAP Health Max 30 and other silver products	Supplemented functional drink	American Biotech Labs, USA	Silver NPs	Antibacterial	[173]
NanoSil-10	Supplemented functional drink	Greenwood Consumer Products, USA	Silver solution	Antibacterial	[173]
MaatShop Crystal Clear Nano Silver	Supplemented functional drink	MaatShop, USA	Silver NPs	Antibacterial	[173]
Silvix3	Supplemented functional drink	Natural Care Products, USA	Silver NPs	Antibacterial and antifungal effects as a surface disinfectant	[173]
Nano Colloidal Silver	Supplemented functional drink	Natural Korea Company Ltd, Korea	Silver NPs	Sterilization and quality control	[173]
Sovereign Silver	Supplemented functional drink	Natural-Immunogenics Corp, USA	Silver hydrosols	Sterilization and quality control	[173]
Nano Silver Sol	Supplemented functional drink	Phoenix P.D.E. Co Ltd, Korea	Silver NPs	Antibacterial activity and sterilization effect	[173]
MesoSilver	Supplemented functional drink	Purest Colloids, Inc., USA	Silver NPs	Highest bioavailability	[173]
Colloidal Silver Liquid	Supplemented functional drink	Skybright Natural Health, New Zealand	Silver NPs	Supports immune system and defense for natural healing	[173]
Utopia Silver Supplements Advanced Colloidal Silver	Supplemented functional drink	Utopia Silver Supplements, USA	Colloidal silver	Sterilization	[173]
Colloidal silver	Food supplement	FairVital, Germany	Colloidal silver consists of small nanoparticles of metallic silver	Colloidal silver particles can be excreted	[173]
Sovereign Silver (8 oz)	Food supplement	Natural-Immunogenics Corp, USA	Actively charged nanocolloidal silver hydrosol	Safely supports immune system	[173]
Silver (16 oz)	Food supplement	Activz, USA	Silver	Support natural healing.	[173]

## Nanotechnology for Food Packaging

Packaging industry contributes largely to the world economy; nearly 55–65% of $130 billion was spent on food and beverage packaging in the USA [19]. In recent years, the application of active and intelligent packaging systems in the muscle-based food products, which are prone to contamination, has increased tremendously. The aim of packaging meat and muscle products is to suppress spoilage, bypass contamination, enhance the tenderness by allowing enzymatic activity, decrease weight loss, and retain the cherry red color in red meats [20]. The advent of nanosensors provides food spoilage or contamination alarm to the consumers by detecting toxins, pesticides, and microbial contamination in the food products, based on flavor production and color formation [21]. Most of the nanoparticles used for packaging in food industry have potential antimicrobial activity, acting as carriers for antimicrobial polypeptides and providing protection against microbial spoilage. Packaging material made of a coating of starch colloids filled with the antimicrobial agent acts as a barrier to microbes through the controlled release of antimicrobials from the packaged material [22].

Nanoparticles are used as carriers to introduce enzymes, antioxidants, anti-browning agents, flavors, and other bioactive materials to improve the shelf life even after the package is opened [23, 24]. Few metals and metal oxide nanoparticles (inorganic nanoparticles), namely iron, silver, zinc oxides, carbon, magnesium oxides, titanium oxides, and silicon dioxide nanoparticles, are widely utilized as antimicrobials and in some conditions as food ingredients [21]. Nanomaterials and their applications in food products are listed in Table 2. The production of reactive oxygen species (ROS) by TiO_2_ is detrimental to pathogenic microbes, making it an effective antimicrobial agent. Enhanced heat resistance, low weight, and mechanical strength, and an increased barrier against O_2_, CO_2_, moisture, UV radiation, and volatiles can be achieved with the use of nanocomposites. Nanocomposites are commonly utilized for packaging and coating purposes [25, 26]. Numerous nanoparticles such as SiO_2_, clay and silicate nanoplatelets, carbon nanotubes, starch nanocrystals, graphene, chitin or chitosan nanoparticles, cellulose-based nanofibers, and other inorganics are filled in a polymeric matrix, making the matrix lighter and fire-resistant with improved thermal properties and low permeability to gases [1]. Nanoparticles (100 nm or less) are included in plastics to enhance their properties. Polymer nanocomposites are thermoplastic polymers composed of 2–8% nanoscale incorporations, such as carbon nanoparticles, nanoclays, polymeric resins, and nanoscale metals and oxides. The extremely reactive nature of nanocomposites over their macroscale counterparts is due to the high surface-to-volume ratio [27]. Silver in the silver zeolite is responsible for the antimicrobial activity via the production of ROS, and the ceramics coated with the silver zeolite are used in food preservation, decontamination of materials, and disinfection of medical products. The sustained antimicrobial performance of silver-based nanocomposite is superior to silver zeolite [28, 29]. Utilization of carbon nanotubes facilitates the elimination of CO_2_ or assimilation of unpleasant flavors. Furthermore, nanoclay in the nanocomposites (bentonite), used in the production of bottles and other food packaging materials, significantly enhances the gas barrier features and thereby inhibits oxygen and moisture from diffusion, drink destabilization, and spoilage of food materials [30, 31].

**Table 2:** List of nanomaterial-based biosensors with their application in food science and food nanotechnology

Nanomaterials	Analyte	Samples	References
Silicon dioxide	Act as food colorant, hygroscopic, anticaking, and drying agent.	Food preservation and packaging	[185]
Titanium dioxide	Used as whitener in dairy products (e.g., milk and cheese)	Food preservation and packaging	[186]
Zinc oxide	Reduces the oxygen flow inside the packed containers	Food preservation and packaging	[186]
Silver nanoparticles	Acts as antibacterial agent, absorbs, and decomposes ethylene in fruit and vegetables	Food preservation and packaging	[187]
Inorganic nanoceramic	Used in cooking (frying)	Food preservation and packaging	[188]
Polymeric nanoparticles	Used as bactericidal and efficient delivery mechanism	Food preservation and packaging	[158]
Chitosan	Used as coating agent for mandarin, strawberries, and fresh fruits	Anti-fungicide	[189]
Gold nanoparticles AuNPs	Integration of DNA or enzymes or antibodies with Au NP Pathogens Glucose	Food storage applications Meat and dairy industries Fruit juice	[190]
SWCNT (single-wall carbon nanotubes)	Integration with biomolecules Fructose Methyl parathion and chlorpyrifos	Wine Honey Phosphate-buffered solution	[191] [192]
MWCNT (multi-walled carbon nanotubes)	Integration biomolecules Paraoxon Fructose	Food industry Phosphate-buffered solution	[193]
CdTe QDs (cadmium telluride quantum dot nanoparticles)	Integration biomolecules	Food industry	[194]
Cu and Au NPs	Pathogens	Surface water	[195]
ZrO2 NPs	Parathion	Phosphate-buffered solution	[196]
Exfoliated graphite nanoplatelet xGnPs	Glucose	Phosphate-buffered solution	[192]
Glyco-NPs	E. coli	Phosphate-buffered solution	[197]
Quantum dots, QDs	*Salmonella typhi*	Chiken carcass wash water	[198]
Gold nanorods	*Pseudomonas*	Sodium chloride	[199]
Silica particles coated with silver shells	*E. coli*	Water	[200]
Au NPs	*Mycobacteriumavium* subsp. *paratuberculosis*	Milk	[201]
CdTe QD	2,4 D (herbicide)	Phosphate-buffered solution	[202]
(CdSe)ZnS core shell QDs	Paraoxon (insecticide)	CH_3_OH/H_2_O (v/v) solvent	[203]
Au NPs	Paraoxon (insecticide)	Glycine buffer	[204]
Fe_3_O_4_ MNPs	Glucose	Acetate buffer solution	[205]
CdTe QDs	Glucose	Phosphate-buffered solution	[203]
CdSe@ZnS NPs	Maltose	Buffer solution	[206]
Silver zeolite	Antimicrobial agent	Preservations, disinfectors, and decontaminants	[29]
Al_2_O_3_, La, Nano	Water purification and soil cleaning	Oxidation of contaminants	[207]
Colloidal metals	Food supplements	Enhanced uptake	[208]
Graphene	Nanoplate-based nanocomposites	Detects contaminants in food	[209]
Cellulose nanocrystals	Biocompatible high water uptake	Food packaging	[210]
Magnetic nanoparticles	Large specific surface area	Pathogen monitoring	[211]
Carbon nanotubes	Optical, electrical, mechanical, and thermal conductivity	Food inspection and vacuum proof food packaging	[16]
Allyl isothiocyanate and carbon nanotubes	Antimicrobial packaging	Enabled effective storage of shredded cooked chicken meat	[212]
Nanolaminates	Food-grade film	Improve the texture properties of foods and serve as carriers	[113]

The inclusion of active nanoparticles into the polymer matrices increases the performance of the food packaging material and provides functional attributes such as antioxidant, antimicrobial, and scavenging which results in the longer shelf life of the packed food products [22]. The utilization of nanocrystals developed by Nanocor (Arlington Heights, USA) in nanocomposite plastic beer bottles is to reduce the loss of CO_2_ and inflow of O_2_ into the beer bottles, like the natural biopolymer-based nanocomposites [32, 33]. The incorporation of clay nanoparticles into the ethylene–vinyl alcohol copolymer and polylactic acid (PLA) biopolymer was shown to improve the oxygen barrier and increase the shelf life of food materials [34]. The organically modified nanoclays incorporated in the polymer matrix provide mechanical strength and serve as a barrier to gases, volatiles, and moisture [35]. Interestingly, PLA bionanocomposite produced from the incorporation of nanofillers into the biodegradable polymer PLA showed more rapid biodegradation than its counterpart PLA without nanofillers. Mechanical, thermal, and barrier properties of the packaging material have been significantly increased by the inclusion of polymer–clay nanocomposites [36]. Obstruction of oxidation, and regulations of moisture migration, respiration rate, microbial growth, volatile flavor, and aromas are greatly influenced by the application of nanotechnology in the packaging industries [19].

Potential antimicrobial activities were reported for the chitosan-based nanocomposite films, particularly silver-containing nanocomposites [37]. Garlic essential oil filled in the PEG-coated nanoparticles can be utilized for the restriction of store-product pests [38]. The shelf life of the food product has been increased efficiently by applying phytoglycogen octenyl nanoparticles included with the Ɛ-polylysine [39]. Application of silicate nanoparticles in food packaging acts as a barrier to gases or moisture and thus decreases food spoilage and drying [40]. Water-based nanocomposites forming 1–2-µm nano-coatings on food packaging materials act as a barrier to oxygen. Nanoemulsions are used in food packaging and decontamination of food packaging equipment. Glycerin included with nanomicelle-based products eliminates pesticide residues from fruits and vegetables and oil/dirt from cutlery. The addition of nanoemulsified bioactive and flavors to beverages does not affect the product appearance [37, 41]. Different food pathogens like gram-negative bacteria are significantly controlled by nanoemulsions. Active and intelligent packaging systems are widely used. Various nanoformulation approaches and their applications in food products are presented in Table 3.

**Table 3:** Different types of nanoformulations and their applications in food industries

Nanostructured materials	Nanoparticles	Methods	Applications	References
Low-density lipoproteins	Fish oil	Microencapsulation	Food additives—mask odor of tuna fish oil	[213]
Biopolymers (proteins or polysaccarides)	Micelles	Microemulsions	Produce glycerides in food products	[214]
Biodegradable biopolymeric NPs	Polylactic acid	Encapsulation	Encapsulate and deliver drugs, vaccines, and proteins	[176]
Liposomes	Phospholipids	Encapsulation	Integrate food antimicrobials for the protection of food products	[215]
Liposomes	Nanoliposomes	Nanoencapsulation	Lipid-based carriers for antioxidants	[216]
Food components integrated with droplets	Colloidal dispersions of droplet	Nanoemulsions	Flavored food products. Milk fortified with vitamins, minerals, and antioxidants	[129]
Polymer matrices reinforced in the nanofillers	Nanoclays, nanooxides, carbon nanotubes, and cellulose microfibrils	Nanocomposites	Biodegradable packaging	[176]
Fine emulsion droplets	Reducing the size of fat globules	Homogenization or micofluidization	High-pressure homogenizers in producing finer milk emulsions	[176]
High-intensity ultrasound waves	Oil and water nanoemulsions	Ultrasound emulsification	To change the characteristics of treated matters	[179]
PLA NPs	Curcumin and quercetin	Encapsulation	As bio-stabilizer	[107]
PLA NPs	Leaf extract	Encapsulation	Developed a greener approach	[107]
Stevioside np	PEG-PLA nanoparticles	Nanoencapsulation	Developed an antidiabetic nutraceutical	[217]
Podophyllotoxin and etoposide	Poly-d,l-lactide nanoparticles (PLA NPs)	Encapsulation	Anticancer activity	[218]
BSA NPs	Tea polyphenols, catechin, and epicatechin	Encapsulation Nanoformulations	Enhance stability and bioavailability Antioxidant potential	[110]
Canola oil	Vitamin E	Nanoemulsions	Nutritional benefits and oxidative stability	[219]

### Active Food Packaging Systems

**Fig. 3:**
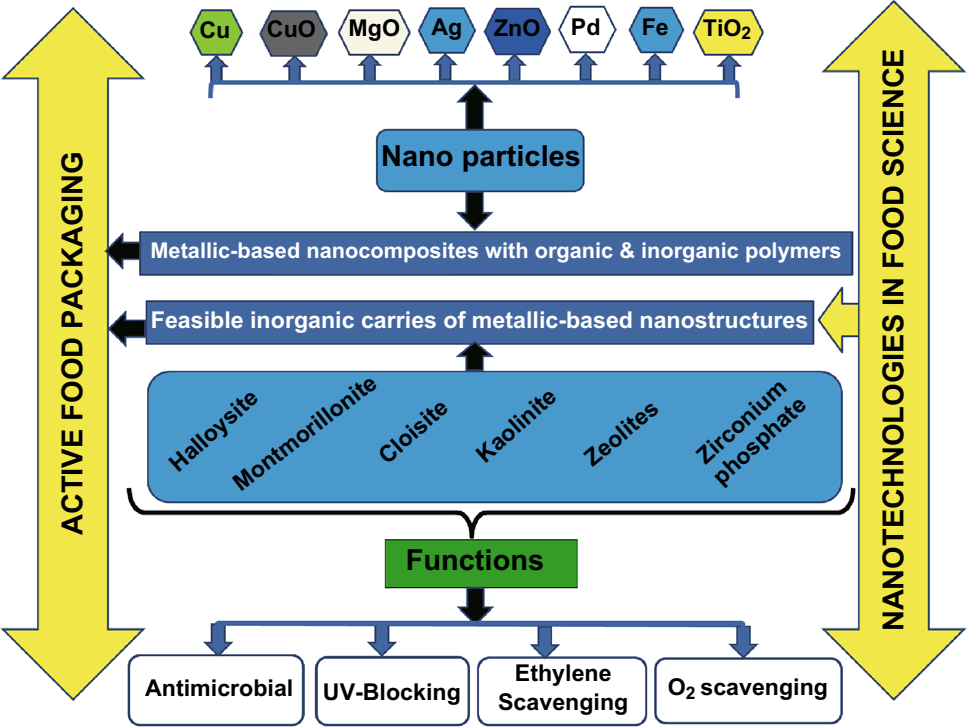
Potential for the development of metallic-based nanocomposites in active food packaging

The active packaging systems consist of moisture regulating agents, CO_2_ scavengers and emitters, oxygen scavengers, and antimicrobials. Active packaging systems are developed depending on the purpose of the storage [42]. For example, overwrap packaging systems are used for short-term chilled storage, while modified atmosphere packaging (MAP) systems, vacuum packaging, MAP systems utilizing 100% CO_2_, and bulk gas flushing are employed for long-term chilled storage. Low-density polyethylene (LDPE) and polypropylene (PP), the commercially used polymeric films for packaging, are inert, are hydrophobic, and have less surface energy [20]. Surface modifications with functional properties and polar groups for the inclusion of antimicrobial substances are essential to eliminate food spoilage [43]. Factors such as lipid oxidation, dehydration, discoloration, and loss of aroma should be considered in the case of processed meats; additives are included in the packaging systems to extend and maintain the shelf life of meat products [20]. Potential for the development of metallic-based nanocomposites in active food packaging is described in Fig. 3.

The MAP is one of the important packaging systems widely operated for the distribution, storage, and maintenance of meat products under cold condition [20]. MAP technology enhances the shelf life and quality of the meat products by replacing the air compassing the meat products with formulated gas mixtures. In general, the non-inert gases such as O_2_ and CO_2_ are used in the MAP technology and their profiles change over time, influenced by factors like the type of product, respiration, materials used for packaging, size of the pack, storage conditions, and package integrity [43]. The uniform dispersion of clay nanoparticles on the transparent plastic film produced by Chemical giant Bayer (Leverkusen, Germany) prevents O_2_, CO_2_, and moisture from reaching fresh meats and other foods. Several patents on the applications of nanomaterials in the food packaging have been filed in the USA, Europe, and Asia, and most of them report the utilization of nanoclays and nanosilver [44]. The inclusion of allyl isothiocyanate and carbon nanotubes into the active packaging systems decreases microbial contamination and color changes, regulates oxidation, and helps in the storage of shredded, cooked chicken meat for 40 days [42].

### Smart/Intelligent Food Packaging Systems

Smart packaging systems respond to environmental stimuli by repairing or alerting the consumer regarding the contamination or the presence of pathogens. Nanoparticles are used in the development of nanosensors to detect food contaminants. Custom-made nanosensors are used for food analysis, detection of flavors or colors, drinking water, and clinical diagnosis [45]. Application of nanosensors in food packaging aids in tracing the physical, chemical, and biological modifications during food processing. Specifically designed nanosensors and nanodevices utilized in smart packaging help in detecting toxins, chemicals, and food pathogens [46]. The intelligent packaging systems with sensors and indicators are also used to track and give information regarding the quality of the packaged foods during storage and transport. Various functional nanomaterials, used as nanosensors and active packaging materials that provide significant mechanical and barrier properties, are potential targeted nutrient delivery systems [47]. With the advent of sensors, sensor-based indicators for integrity, freshness and time–temperature monitoring and radio frequency identification were used in the meat industry [20]. It has been reported that smart or intelligent packaging retains the food quality during distribution. The response to modifications associated with the internal or external environmental stimuli is registered by the specific sensor. Integrity (package integrity determination), freshness (quality of the packaged products), and time–temperature (time and temperature dependent changes) indicators are commonly used in the food packaging applications. These indicators are monitored throughout the production and distribution chain in order to maintain the quality and enhance the shelf life of products. Barcodes developed with the help of nanoparticles, called nanobarcodes, can be used as ID tags [48].

Nanosensor application in packaging provides details of enzymes generated during the degradation of food compounds that makes food unsuitable for human intake. Packaging improves the shelf life of the food products by preventing air and other enzymes from entering and decreases the use of artificial preservatives. It also helps in the elimination of the ripening hormone known as ethylene to enhance the shelf life of food products [49]. The nanosensors in the smart packaging systems are used for the detection of gases, chemical contaminants, aromas, temperature and light intensity, pathogens, or the products of microbial metabolism [50]. Analytical techniques such as GC/MS, portable headspace O_2,_ and CO_2_ gas analyzers are available to investigate the gas phases in the MAP products. However, these methods have certain demerits; optical sensor-based approaches are more effective than these methods in real-time packaging processes or large-scale usage [20, 43]. Food rotting is a major concern in the food industry; it is caused mainly by bacteria that result in the release of unpleasant odor, which may be difficult to detect with human nose, and sometimes rotting food may lead to poisoning. In order to detect the odors generated due to food poisoning, highly sensitive biosensors are required [51]. For example, the device electronic nose functions like a human nose which utilizes sets of chemical sensors attached to a data processing system. Methods to determine chemical and physical characteristics of pears and fruit odors using the electronic nose signal have been reported [52]. Interestingly, electronic nose can be used to detect variations in the aroma of strawberry fruit, osmotic dehydration, and the quality of milk during processing. This device is used for a highly accurate determination of volatiles and monitoring the quality control processes in food industry. Nanosensors were applied in the European project GOODFOOD (2004–2007) for food safety and quality control applications [53].

## Mechanism of Nanoparticle Activity

Common factors such as product nature (formulation), processing conditions (intrinsic factors), type of package and storage and distribution crucially affect the shelf life of a food product [54]. Intrinsic factors like water activity, pH, microbes, enzymes, and the level of reactive compounds can be regulated by using specific raw materials and ingredients and appropriate processing parameters. Temperature, total pressure, light, partial pressure of various gases, relative humidity, and mechanical stress (human handling) are the common extrinsic factors that influence the rate of degradation reactions during food material storage [55]. It is worth noting that the microbial growth mostly occurs on the surface of the perishable foods, including muscle-based foods, and therefore, utilization of antimicrobial packaging efficiently controls the microbial growth compared to the application of antimicrobials as food additives. Furthermore, antimicrobial packaging effectively interacts with the food product as well as the environment [56]. Nanoparticles are widely used in the packaging systems due to their potential antimicrobial properties. Most of the nanoparticles produce ROS, thus damaging the microbes present on the surface of food and packaging materials. Antimicrobial nanoparticles, namely Cu, CuO, MgO, Ag, ZnO, Pd, Fe, and TiO_2_, or nanoemulsions/nanoencapsulations enclosing natural antimicrobial substances that can be adhered to via electrostatic, hydrogen bonding, and covalent interactions are developed to produce antimicrobial packaging systems. Several chemical modifications and deposition processes are being used for the attachment of silver NP onto the surface of the plastic substrate that facilitates slower release of silver ions to reduce their inclusion in the food [43].

**Fig. 4:**
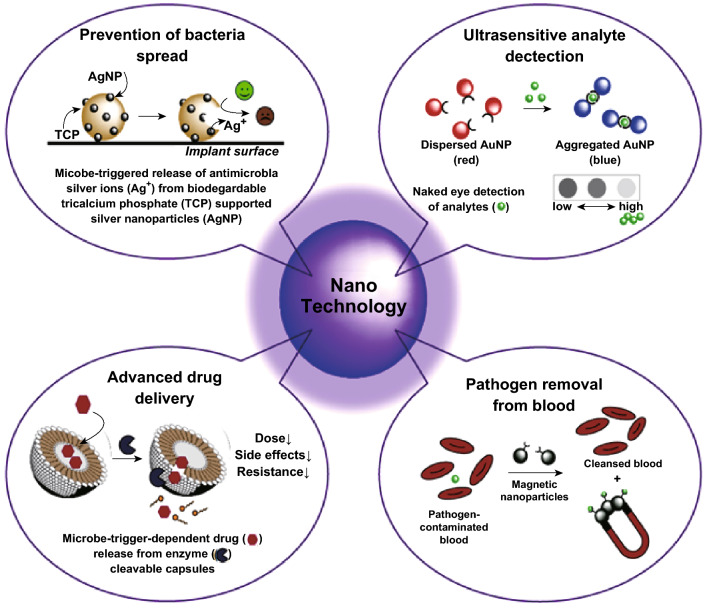
Examples of nanotechnology-based solutions for detection and prevention of microbial infections. This figure was modified and adopted from Ref. [256] with permission

The potential surface charge of engineered water nanostructures (EWNS) can deactivate *Salmonella enterica, Escherichia coli*, and *Listeria innocua* effectively on the surface of stainless steel and on tomato without influencing the sensory quality of food, operating via ROS production. The degradation of EWNS results in the formation of water vapor, hence lowering the risk of hazardous environmental problems [57]. The inorganic (ZnO, TiO_2_, and Ag) and organic (chitosan and essential oil) nanomaterials are also used for food product preservation. Since polymer matrices control the release of active components, they regulate the function of nanocomposites. Polymers such as polyolefins, nylons, ethylene–vinyl acetate (EVA) copolymer, polyethyleneterephthalate (PET), polystyrene (PS), polyamides, and polyimides have been used for nanocomposite production [58]. For example, silver NPs immobilized in cellulose and collagen sausages casings showed potential bactericidal activity against *E. coli* and *Staphylococcus aureus,* but they were not harmful to humans and the environment [55]. Such superior antimicrobial activity against *E. coli* and *S. aureus* had been demonstrated with silver–polyamide nanocomposites, where the antimicrobial efficiency lasted for 28 days. The antimicrobial nature of the nanocomposite is significantly affected by the characteristics of the polymer and NP [59]. Various types of nanoparticles that could help to prevent, detect, or treat bacterial infections like silver, gold, and tiny magnetic particles help to trigger or capture bacterial pathogens and help to prevent bacterial spread in hospitals and human body, providing an attractive way to rapidly detect bacterial biomolecules in a point-of-care compatible setting [43, 59]. The details about different nanotechnology-based solutions that could help to prevent, detect, or treat bacterial infections are presented in Fig. 4.

The LDPE films coated with silver NPs using a layer-by-layer method have a significant antimicrobial potential against *S. aureus* (gram-positive) and *Pseudomonas fluorescens* (gram-negative) [60]. Similarly, significant antimicrobial functions against *L. monocytogenes, E. coli* O157: H7, *S. aureus*, and *S. typhimurium* were observed in the chitosan–silver nanocomposite prepared by the solvent casting method [37]. ZnO-encapsulated halloysite–polylactic acid nanocomposite comprising packaging films exhibited enhanced mechanical and antimicrobial activities [61]. Pathogenic bacteria species such as *E. coli, Listeria monocytogenes,* and *P. aeruginosa* residing in the meat products were deactivated by the LDPE/ZnO^+^Ag nanocomposites. In addition, the application of these nanocomposites enhanced the shelf life of the chicken breast fillets and slowed down the bacterial growth and lipid oxidation. It was reported that the improved quality of lemon with lower total soluble solids and punch force, enhanced titratable acid, firmness, and peel shear forces were found when they were coated with the chosen clay nanocomposites during cold storage [62].

The storage conditions and duration also influence the antimicrobial activities of the nanocomposite films. For example, the stability and antimicrobial activity of pullulan films incorporated with NP (silver or ZnO NPs) and oregano or rosemary essential oils were studied at various temperatures (4, 25, 37, and 55 °C) for 7 weeks against the common food pathogens such as *L. monocytogenes* and *S. aureus* [63]. Their results illustrated that the antimicrobial potential of the pullulan nanocomposite films was maintained at low temperature (< 25 °C), and reduced greatly at > 25 °C. Some studied showed that the migration of Ag from the Ag/PVC films to the chicken meat was least (8.85 mg/kg or 0.84 mg dm^−2^), below the legal migration limits provided by the European Union for plastic films [64]. Food packaging with low silver concentration, having an enhanced and stable bioavailability is a challenge for the application of silver in food packaging. The citrate-mediated silver complex is the most commonly used standardized silver formulation for antimicrobial purposes [65].

### Antimicrobial Properties of Nanoparticles

Several nanoproducts are available in the market to control the microbial growth. For instance, the four crucial food-related pathogens such as *E. coli* O157: H7, *S. typhimurium, Vibrio parahaemolyticus,* and *L. monocytogenes* were effectively inhibited by the nanosilver product known as NanoCid^®^ L2000 (Nano Nasb Pars Company, Tehran, Iran) [66]. Nanotechnology Consumer Product Inventory has listed many nanomaterial-related antimicrobial disinfectants. Most of the antimicrobial products found in the list contain nanosilver as the main antimicrobial agent [67]. The antibacterial activity of silver nanoparticles alone and the silver NP embedded in the carboxymethylcellulose film showed that AgNPs embedded in the carboxymethylcellulose film were more potent as bactericidal than the AgNPs alone, which was suggested that antibacterial activity of AgNPs can be used food packaging [68]. Nanoengineered surfaces (antimicrobial coatings) are one of the efficient agents to suppress the growth of biofilms and enhance the quality and safety of the food. The nanoscale silver, TiO_2_, and ZnO or nanoscale topography is used for surface cleaning in the food industry. The biocontamination problems existing in poultry farming, food processing, and food transportation were effectively controlled by the UV-C ultraviolet light-activated TiO_2_ [69]. The pathogen transmission is mainly through air and may be involved in poultry meat contamination at different stages of slaughtering and processing [50]. The incorporation of microbicidal materials such as silver and other metals in the nanofiber mats revealed a significant antimicrobial potential [69]. Nanoenabled membranes, nanophotocatalysts, and nanoadsorbents are used for the purification of water in the wastewater treatment [70].

### Synergistic Antimicrobial Effects of Nanoparticles

The combinatorial use of two or more nanoparticles provides a synergistic effect exhibiting potent antimicrobial activity compared to a single nanoparticle. Silver NPs combined with titanium dioxide and carbon nanotubes effectively combat *E. coli* and *Bacillus cereus* spores, respectively [71]. *B. cereus* spores present on the surfaces of aluminum and polyesters were destroyed by the silver-doped TiO_2_ NP. The molds and airborne bacteria caught in the air filters are destroyed by the silver-doped TiO_2_ NP [72]. Enhanced antimicrobial activity against *E. coli* and *S. aureus* was achieved by the stabilization of silver NP with SDS or PVP. Silver NP coatings have been used on the surfaces of refrigerators and storage containers [73]. Several food-related pathogens, such as *Vibrio parahaemolyticus, Salmonella choleraesuis*, and *Listeria monocytogenes,* are shown to be affected by the UV-activated TiO_2_ NP Photoactivation of TiO_2_, reportedly caused biocidal activities against toxic food microbes [74, 75].

### Synergistic Antimicrobial Activity with Natural Derivatives

Several studies have shown the synergistic antimicrobial activities of various nanoparticles (silver, gold, zinc, chitosan, platinum, iron, copper, carbon nanotubes) with the essential oils (natural derivatives) [76]. Researchers have formulated an essential oil (EO) droplet emulsified with gold NP; also they utilized NP for the encapsulation of peppermint EOs and cinnamaldehyde [77, 78]. Similarly, thymols containing EO of *Lippia sidoides* were nanoencapsulated in the chitosan–gum NPs and are used in chemical, pharmaceutical, and food industries [79]. Magnetic nanofluid was produced by fusion of EOs and iron oxide NPs [80]. Oregano EO nanoencapsulated with the chitosan NPs was studied for its antimicrobial activity as well as the releasing pattern of Eos [81]. The antimicrobial activity of thymol was increased when encapsulated with the zein-sodium caseinate NPs [82]. The antibacterial and antifungal activity of the EOs is increased through nano-complex of various types of NPs. The eugenol and cinnamaldehyde incorporated in the poly (D,L-lactide-co-glycolide) (PLGA) NPs were shown to have potent biocidal activity against Salmonella and Listeria [83]. Similarly, the combinatorial preparation of liposome-based NP and *Origanum dictamnus* essential oil was highly effective in controlling gram-positive and gram-negative bacteria [84]. Several essential oil derivatives of *Santolina insularis* such as γ-terpinene, carvacrol, p-cymene, thymol, and their combination with phosphatidylcholine liposome NPs were prepared by researchers, and they have been effective in controlling the growth of microbes [85].

The researchers reported that the encapsulation of essential oil derivatives such as thymol and carvacrol within the zein nanoparticles through liquid–liquid dispersion method demonstrated a strong antioxidant and antimicrobial activity against *E. coli*. Nanoencapsulation enhances the activity of essential oil against microbes, and the polymeric nanoparticles, liposomes, and nanoemulsions are employed for this purpose; this can be used to regulate the release of drug molecule [86, 87]. Potential biocidal activity of EO encapsulated by chitosan/cashew gum nanoencapsulation was found against *Stegomyia aegypti* larvae due to the slow and sustained release [88]. The gram-positive bacterial growth was effectively suppressed by the thymol encapsulated in zein nanoparticle compared to thymol only [89]. Carvacrol, a monoterpenic phenol produced by aromatic plants, increased antimicrobial activity when nanoencapsulated with polylactic glycolic acid [90]. The gold nanoparticles linked with the vancomycin substituent showed more toxicity toward vancomycin-resistant bacteria [91]. Phytoglycogen NP coupled with nisin showed improved antimicrobial action against *L. monocytogenes* [92]. The application of pullulan film containing essential oils (2% oregano, 2% rosemary) and NPs (100 nm Ag, 110 nm ZnO) to fresh turkey, raw beef, or processed turkey deli meat resulted in the suppression of *L. monocytogenes, S. typhimurium, S. aureus*, and *E. coli* O157:H7 for more than 2 weeks when vacuum-packaged and stored at 4 °C [93]. PLA/CEO/β-CD nanofilms were developed by the inclusion of cinnamon EO-β-cyclodextrin inclusion complex (CEO/β-CD-IC) into the PLA nanofibers using electrospinning technique and showed potential antimicrobial functions against *S. aureus* and *E. coli*. In addition, the shelf life of fresh pork was increased to 8 days compared to control samples which had a shelf life of 3 days [94]. Nanoencapsulation of essential oil enhanced their physical stability and bioactivity, reduced the volatility and toxicity, and protected it from environmental interactions with oxygen, light, pH, and moisture [95]. Therefore, the combination of essential oils and nanoparticles significantly increased the antimicrobial properties as they complemented each other against various pathogens; this would be an ideal strategy to constrain the multidrug resistant microbes (MDR). The emergence of MDR pathogens results in increased rate of morbidity and mortality, emphasizing the need for alternative natural drugs. Moreover, application of nanotechnology to the natural drug product formulation may ensure slow and sustained release of drugs to combat MDR microbes [76–80].

## Applications in Nutraceutical Delivery and Bioavailability

**Fig. 5:**
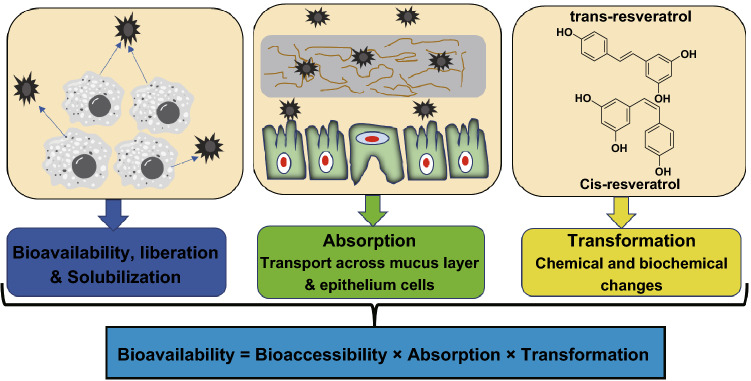
Oral bioavailability of bioactive compounds controlled by three major steps: bioaccessibility, absorption, and transformation. This figure was modified and adopted from Ref. [257] with permission

Bioactive substances present in food provide immunity and protect against diseases. Although most of the food items possessed higher concentrations of bioactive molecules, their potency was low. It is mainly due to low bioavailability, lower solubility, and stability in the gut, decreased permeability, and retention time in the intestinal tract [96]. Nanomaterials usually consist of a wide surface area per unit mass and decreased particle dimension which enhances the biological activity, bioavailability, and solubility of the encapsulated food materials [16]. Nanosized iron and iron/zinc materials used in the nutraceutical deliveries enhanced the bioavailability and reduced the color changes in the final products. The bioavailability of most of the vitamins (A, D, and E) and bioactive compounds such as curcumin, carotenoids, conjugated linoleic acids, coenzyme Q_10,_ and ω-30 fatty acids is low or unstable after intake [97]. Low bioavailability is due to the physicochemical and physiological parameters such as bioaccessibility, absorption, and transformation. In general, the low bioavailability, solubility, and stability of most of the bioactive molecules such as antioxidants, vitamins, micronutrients, polyphenols, carotenoids, and food ingredients can be enhanced with the help of nanotechnology specifically with nanoformulations [16]. Most of the biologically active substances used in treatment of diseases are hydrophobic in nature having least bioavailability. Nanotechnology-based delivery systems are used to enhance the bioavailability and targeted delivery of natural bioactive compounds (Fig. 5).

**Fig. 6:**
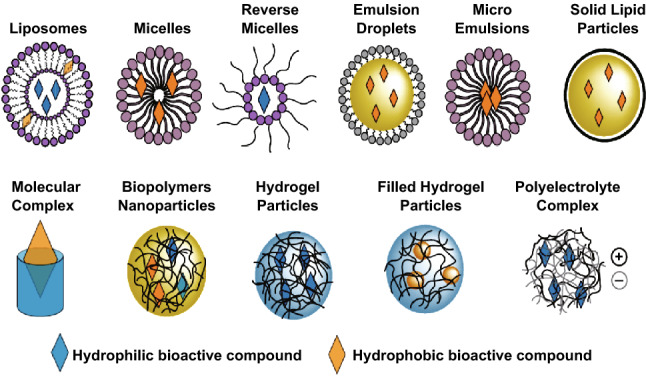
Examples of effective micronutrient delivery system for food manufactures. This figure was modified and adopted from Ref. [101] with permission

Nanoparticles are also made up of natural food-grade macromolecules such as proteins, surfactants, polysaccharides, lipids, and phospholipids. Composite nanoparticles are produced by different combinations of these food-grade ingredients, such as lipid core with a protein referred to as “nanoemulsions” and lipid droplets inserted in the biopolymer microspheres [95]. The efficiency, utilization, and stability of the bioactive food materials can be enhanced with the help of these food-grade nanoparticles due to their encapsulating nature, protection, and release of the bioactive food constituents [98]. Nanonutraceuticals are a combination of nutrition and pharmaceuticals in which the dietary supplements, bioactive substances, functional foods, and herbal products are produced via nanoformulation approach [21]. Different methods were employed for the delivery of nutraceuticals. Nanotubes, nanofibres, fullerenes, nanosheets, nanowhiskers are delivered via various vehicles such as liposomes, cubosome, microemulsions, solid lipid nanoparticles (SLNs), biopolymeric nanoparticles, nanosensors, monolayers, microgels, and fibers [99]. It is crucial to understand the advantages and the significant toxicity of nanocarrier systems in food products. For development of effective micronutrient delivery system for food manufactures, a great advancement in design and fabrication of various food-grade nanoparticles is done by recently with the addition of notable applications [100]. Colloidal delivery-based foods (excipient foods) can improve the bioavailability of the food, although they do not possess any biological activity themselves. The absorption of the bioactive agents into the systemic circulation increases, resulting in enhanced bioactivity while providing powerful health benefits. In order to enhance the bioaccessibility, absorption, or transformation profiles of bioactive compounds in the gastrointestinal tract (GTI), the composition and structure of excipient foods are specifically designed [101, 102]. There are a number of nanoparticle-based delivery systems to improve the bioavailability of the food with suitable encapsulation of micronutrients presented in Fig. 6. The applications of nanotechnology in nutraceuticals and pharmaceuticals which was discussed by many researchers in their studies are shown in Table 4.

**Table 4:** Utilization of nanoparticles in nutraceuticals and pharmaceuticals with their applications

Nutraceuticals			Pharmaceuticals		
Nanoparticles	Applications	References	Nanoparticles	Applications	References
Nanoliposomes	Specific delivery of nutraceuticals	[218]	Polylactic acid	To deliver drugs, vaccines, and proteins	[220]
Alginate and chitosan	Target delivery of lutein, β-carotene; lycopene; vitamins A, D, and E3; co-enzymeQ 10; and omega-3-fatty acids	[221]	Nanoencapsulated probiotics	Selectively delivered to gastrointestinal tract and modulate immune response	[119]
Synthetic and natural polymers	Used in food packaging	[222]	Podophyllotoxin and etoposide	Anti-disease particles	[223]
Nanosize ingredients and additives	Enhances taste, absorption, and bioavailability	[224]	Antibodies attached to nanoparticles	To detect chemicals or food-borne pathogens	[225]
Incorporated carriers consist of lycopene, beta-carotenes, and phytosterols	To avoid the accumulation of cholesterol	[176]	Biodegradable nanosensors	Monitors temperature, moisture, and time.	[226]
Nanoselenium	Green tea helps in uptake of selenium	[227]	Nanoclays and nanofilms	Prevent spoilage and oxygen absorption	[228]
Functional ingredients nanodelivery system	Increases the functionality and stability of the foods	[229]	Cellulose nanocrystal composite	Drug carrier	[230]
Nanocochleates	Stabilize micronutrients and enhance the nutritional value	[208]	Nanoparticles of silver, magnesium and zinc	Antimicrobial and antifungal surface coatings	[231]
HydraCel	Improve absorption of water and other nutrients in the blood and decrease the surface tension	[176]	Carbon nanotubes	Carrier for gene and peptide delivery. Enhances the solubility and penetration	[232]
α-lactalbumin (nanoencapsulated)	Carrier of nutrients	[21]	Dendrimer	Controlled and targeted the delivery of bioactive compounds to macrophages and liver	[233]
Casein micelles	Delivers sensitive products	[234]	Liposomes	Active and passive delivery of gene, protein, and peptide	[128]
Dextrin’s and curcumin	Bioactive products	[235]	Metallic nanoparticles	Enhanced radiotherapy and highly sensitive diagnosis	[236]
Nanoparticle-mediated gene or DNA transfer	Development of insect-resistant plants, nanofeed additives, food processing, and storage	[21]	Nanocrystals quantum dots	Multiple color imaging of liver. Labeling of breast cancer marker, HeR 2 surface of cancer cells	[237]
Tailor-made nanosensors	Food analysis	[176]	Polymeric micelles	Active and passive drug delivery	[238]
Nanocapsules	Improve bioavailability of nutraceuticals (cooking oils)	[239]	Polymeric nanoparticles	Excellent carrier for controlled and sustained delivery of drugs	[240]
Nanoencapsulate	Flavor enhancer	[241]	Mesoporous silica	Delivery of drugs	[242]
Gelated nanotubes and nanoparticles	Viscosifying agent	[243]	Biosensor	Understanding living cells	[244]
Nanocapsule infusion of plant-based steroids	Replace meat cholesterol	[245]	Fluorescent nanoparticles	Cell labeling	[246]
Nanoemulsions	Availability and dispersion of nutrients	[247]	Magnetic nanoparticles	Sensors	[212]
Selective binding of nanoparticles	Removes chemicals or pathogens	[248]	Colloidal gold nanoparticles	Used to monitor cells	[249]
Electrochemical nanosensors	Detect ethylene in food packaging	[176]	Superparamagnetic iron oxide	Cancer detection	[250]
Silicate nanoparticles	More heat-resistant films	[251]	Metal nanocluster	Organosols	[252]
Nanosize powders	Increase absorption of nutrients	[250]	Metal nanocolloids	Hydrosols	[252]
Nanoencapsulation of nutraceuticals	Better absorption and stability of delivered targets	[112]	Magnetic fluids	Sensors	[253]
Nanocochleates	Nutrients are efficiently delivered without affecting color and taste	[181]	Fullerenes (carbon 60)	Potential application in medicinal field	[249]
Nanodroplets (vitamin sprays)	Better absorption of active molecules	[254]	Raw nanomaterials	Drug encapsulation and bone replacement	[253]

### Nanoemulsions

**Fig. 7:**
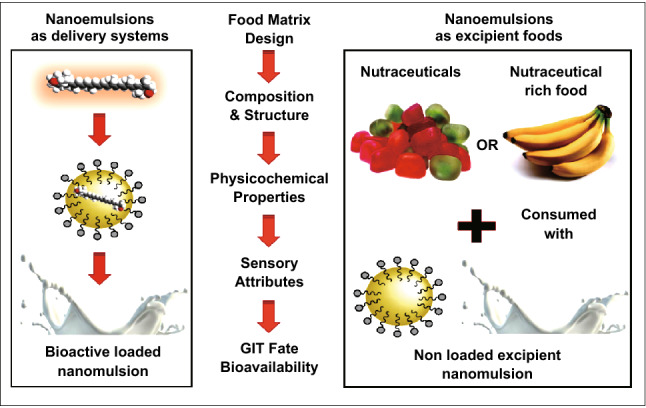
Schematic diagram showing the delivery of bioactive components in food matrix. This figure was modified and adopted from Ref. [257] with permission

Nanoemulsions are a colloidal particulate system with oil-in-water emulsions characteristics having a very small droplet size that varies from 10 to 1000 nm and containing solid spheres with amorphous and lipophilic surfaces. The nanoemulsions act as excellent carriers for various bioactive compounds with enhanced properties compared to conventional emulsions, providing with excellent properties like high optical clarity, physical stability, and enhanced bioavailability [103]. The small size of nanoemulsions helps to produce or have large surface area which can be very important for strong interaction with various bioactive compounds transported in the gastrointestinal tract. Also, the nanoemulsions show higher digestion rate compared to conventional emulsions as they are having more binding sites available amylase and lipase digestive enzymes in the gastrointestinal tract [104]. Moreover, these nanoemulsions are significantly helped in rapid transfer of naturally occurring hydrophobic bioactive compounds present in functional foods into the oil droplets. Various functional foods are the types of foods which significantly help to produce energy and ameliorate the human health problems [103]. Some examples of functional food products which are already available for human beings are cereals with vitamins, minerals and ω-3 fatty acids, curds or yogurts with probiotics, milk products fortified with vitamin D, fruit juices enriched with various metal ions like iron and calcium, and breads fortified with phytosterols [105]. The concept of development of functional or healthier food product has gained more importance to optimize and enhance various natural bioactive compounds as food for amelioration of intrinsic health properties and bioavailability. In this context, excipient foods have been introduced as foods that are able to improve the bioactivity of foods co-ingested with them (Fig. 7) [102, 106]. The nanoemulsion-based approach effectively increases bioavailability of biologically active compounds as their structures, compositions, and properties can be regulated. Emulsion-based systems are prepared from the emulsifier-coated oil droplets dispersed in water phase. The conventional emulsion-based systems are larger in size (oil in water, *d* > 100 nm) while the latest nanoemulsions are smaller in size (*d* < 100 nm) [24, 96]. The nonpolar domains containing a mixed micelle phase are larger in number and harbor all released hydrophobic bioactive compounds, thus enhancing their bioaccessibility [97]. The inclusion of isolated bioactive compounds into the emulsion-based delivery systems leads to enhanced bioavailability. However, the enhanced bioavailability of bioactive compounds present in whole foods can be achieved by incorporating them into emulsion-based excipient systems (EES) [96, 97]. Polyphenols are the naturally derived secondary metabolites possessing various health benefits. The stability and oral bioavailability of the epigallocatechin gallate and curcumin were enhanced by the nanoemulsion method, and the nanoemulsion was used to enhance the yellow color pigment in turmeric [104]. The major applications of nanoemulsions include curing and treatment for enzyme replacement therapy in the liver, infection of the reticuloendothelial system, cancer prevention, and vaccination [103].

### Nanoencapsulation

Encapsulation of bioactive nutraceutical molecules using nanoformulations increased their bioavailability and biodistribution [107]. Nanoencapsulation is a process in which the substances are packed in tiny structures, either by nano-structuration, nano-emulsification, or nanocomposites that facilitate controlled release of the core. Different types of nanoencapsulations (nanoparticles, liposomes, nanospheres, micelles, nanocochleates, and nanoemulsions) have been employed depending on the requirement. They can be used as nutritional supplements, to conceal unpleasant taste, improve the bioavailability, and permit efficient dispersion of insoluble supplements without the requirement for surfactants or emulsifiers [99, 108]. The PLA-based nanoparticles were used as a stabilizer in the nanoencapsulated curcumin and quercetin in turmeric extract [16]. Stevioside nanoparticles, nontoxic natural noncaloric sweeteners, were used as antidiabetic nutraceutical agents [109]. The stability and bioavailability of the polyphenols (catechin and epicatechin) in tea were enhanced by encapsulating them in BSA nanoparticles [110]. The nanoformulation of bioactive components which results in their slow release retains the antioxidant potential and enhances the effectiveness of the bioactive molecules. Most of the natural phytochemicals are sensitive to different environments [111]. The entrapment of biologically active components (vitamins, antioxidants, phytochemicals, proteins, lipids and carbohydrates) within the nanoparticles not only gives protection, but also improves function and stability of the bioactive. These nanocapsules when dissolved release the active ingredients like the normal food [112, 113]. The encapsulation of curcumin in hydrophobically modified starch results in increased anticancer activity [114]. Nanocoating can be used as vehicles for functional ingredients during nanoencapsulation, whereas the nanocapsules can be designed to include nanoadditives, antimicrobials, and detoxifying agents (e.g., mycotoxin binding) in the animal feeds. Some of the compounds (e.g., octenyl succinic anhydride-Ɛ-polylysine) are bifunctional molecules which can be either utilized as surfactants or emulsifiers and can be used in the encapsulation of bioactive compounds or drugs or antimicrobials [115, 116]. Interestingly, nanoencapsulation using lipid molecules increased the antioxidant potential through enhanced solubility and bioavailability and eliminated the undesirable interactions with other food constituents. Nanoliposomes, nanocochleates and archaeosomes are commonly employed lipid-based nanoencapsulation systems. Nanoliposomes are potentially used as cargos for nutrients, enzymes, food antimicrobials, and food additives [117]. Nanoencapsulation of probiotics was also reported previously. The live mixtures of bacterial species supplemented in the food are known as probiotics. The common probiotic foods are cheese, fruit-based drinks, yogurts, and yogurt-type fermented milk and puddings. Encapsulation of these ingredients increases the shelf life of the product. The designer probiotic bacterial species produced using nanoencapsulation technique can be introduced into specific regions of the GI tract where they bind to the specific receptors. These nanoencapsulated designer probiotics may function as de novo vaccines, with the potential to regulate immune responses [118, 119].

### Mixed Nanoparticle Delivery System

Several studies demonstrated the encapsulation of a bioactive agent using a single type of nanoparticle in the food industry. A mixed nanoparticle delivery system (two or more types of nanoparticles with various functional characteristics) can be more useful for certain applications. It was reported that the encapsulation of chemically sensitive (labile), hydrophobic, biologically active substances using protein nanoparticles followed by mixing with lipid nanoparticles is highly advantageous; protein nanoparticle provides protection to the bioactive; and lipid nanoparticles attribute a source of digestible triglycerides (TGs) that in turn enhances the solubility of the bioactive components in the gastrointestinal tract [96, 120]. The triglycerides get hydrolyzed to free fatty acids and monoglycerides in the small intestine. These hydrolyzed products combine with the bile acids and phospholipids to produce mixed micelles that dissolve the hydrophobic bioactive substances once they are liberated from the encapsulated protein nanoparticles. Few studies have reported the fabrication of either curcumin-loaded or tangeritin-encapsulated zein nanoparticles with antisolvent preparation followed by mixing with lipid NP using microfluidization [121, 122]. They found that enhanced solubilizing ability of the mixed micelle phase significantly improved the bioaccessibility of the hydrophobic bioactive molecules. Nanoparticle clustering is a mixture of positively and negatively charged suspension nanoparticles of proportions resulting in the formation of highly viscous solutions or gels (NP clusters) [123, 124]. The pH and ionic strength strongly influence the interaction between the oppositely charged particles. This concept is used to produce less calorie food products with enhanced or novel textural features. Trojan Horse NP is the entrapment of nanoparticles in larger particles (hydrogel beads) leading to enhanced functional features. In this delivery method, nanoparticles filled with biologically active substances can be trapped inside the larger particles and are liberated when they reach the site of action [125]. It was utilized to encapsulate nanoemulsions in larger particles (hydrogel beads) where the release depends on the environment [96]. In the upper GI tract, Trojan Horse nanoparticle systems provide protection to oil droplets from lipid digestion and then allow their release within the colon [126]. In addition, digestion rate of lipid droplets in the small intestine and bioaccessibility of encapsulated hydrophobic bioactive substances can be regulated with the Trojan Horse nanoparticle systems [125]. The major achievement of this system is the delivery of the bioactive substances to various regions of the gastrointestinal tract. Nanosized, self-assembled liquid structures are called as fortifying nano-vehicles where the expanded micelles (< 30 nm) are used for the targeted nutraceuticals such as beta-carotene, lycopene, isoflavones, coenzyme Q_10_ (CoQ_10_), phytosterols, and omega-3 fatty acids [127].

### Environment-Specific NP-Mediated Delivery

The development of diagnostic sensors and controlled release delivery systems requires nanoparticles which can alter their characteristics with response to the specific environmental stimulations including ionic strength, pH, enzymatic activity, and temperature. Several studies have been carried out to develop NP that can change their properties according to the environment. For instance, bioactive molecules carrying lipid nanoparticles were broken down immediately upon contact with lipase and bioactive molecules were released under simulated GIT conditions [96]. The pancreatic lipase releases the bioactive substances in the small intestine, while the bioactive substance-filled protein nanoparticles were readily broken down in the stomach or small intestine containing proteases. Whey protein-loaded riboflavin, zein nanoparticle-loaded curcumin, and resveratrol are some of the bioactive-loaded protein nanoparticles [96]. In the pharmaceutical industry, pH-sensitive nanoparticles have been used to deliver anticancer molecules to the oncogenic tissues.

### Natural Carriers

Nano-vehicles were developed to carry out specific functions. Natural nanocarrier of nutrients such as casein micelle is used for the delivery of hydrophobic, bioactive substances. For hydrophobic nutraceutical delivery in clear acid beverages, β-lactoglobulin–pectin nanocomplexes and core–shell NP built from heat-aggregated β-lactoglobulin and nanocoated using beet pectin for bioactive molecules delivery were generated [128, 129]. Milk proteins such as lactoferrin or bovine serum albumin-fused NP were used for the potential drug delivery across the blood–brain barrier, in vivo. Nanoparticles were designed for targeted delivery to specific region, organ, or tissues. For example, targeted delivery to gastric cancer (β-casein NP), intestine (BSA NP coated with the fatty acid), and colon (Maillard conjugates of casein and resistant starch) was achieved with these applications [21]. Soy lecithin used to produce aqueous nanodispersions acts as a carrier of hydrophobic bioactive, including fat-soluble vitamins. A sevenfold higher absorption of CoQ in the intestine was observed in the nanodispersion method compared to conventional powder formulations [130]. Colloidosomes are self-connected tiny capsules forming a hollow shell in which bioactive or any other substances can be filled. Beta-carotene encapsulated in nanolipid carriers was designed which permits the hydrophobic β-carotene to easily disperse and stabilize in beverages [131]. Nanocochleates are nanocolloid particles built mostly from lipids (75%) that encapsulate the micronutrients and provide stability, protection, and food with improved nutritional values. Starch like nanoparticles significantly improved the stability of the oil-in-water emulsion by preventing lipid oxidation [132].

## Nanomaterials in Diagnostic Applications

Although the conventional molecular diagnostic methods are shown to have higher sensitivity and reproducibility in the detection of pathogens as well as their products (toxins), they are not used in many places due to the requirements for sophistication, high-cost instrumentation, and trained technicians. The unique magnetic, electrical, luminescent, and catalytic activities of nanomaterials are used for the development of rapid, sensitive, and low-cost diagnostic assays for the detection of microbial pathogens. With the application of nanosensors, microbial pathogen detection is rapid, sensitive, accurate, and low labor-intensive. In general, NPs are highly reactive compared to their large-sized particles; hence, it is worth noting to study their possible toxicity in living systems [133, 134].

### Liposomes

The detection of bacterial toxins such as botulinum, tetanus, and cholera was achieved with the help of engineered GT1b or GM1 ganglioside-bearing liposomes which are in the range of ∼ 120–130 nm, and they recognize the target toxins. Fluorescent-labeled (rhodamine dye) liposomes were able to detect the lowest concentrations of toxins (1 nM) using fluoroimmunoassay [135].

### Carbon Nanotubes

Galactose biofunctionalized single-walled nanotubes (Gal-SWNTs), used to detect *E. coli* O157:H7 containing galactose binding surface proteins, revealed a strong aggregation due to their multivalent interactions [136]. It was also reported that application of SWNT-mediated potentiometric aptamer biosensor was used for faster and label-free detection of live bacterial cells; 6 cfu mL^−1^ in milk and 26 cfu mL^−1^ in apple juice [137].

### Gold Nanoparticles (Au NPs)

Gold nanoparticles are considered suitable for the adsorption of biomolecules without losing their biological functions, mainly due to their large surface-to-volume ratio and unique physical and chemical properties. *Staphylococcus aureus* cell membrane protein (protein A) was detected by the AuNP-anti-protein A antibody conjugate immobilized on the immunochromatographic strip. This device functions in a rapid (< 10 min) and highly sensitive (25 ng mL^−1^) manner for the detection of protein A [138]. The detection of DNA from pathogenic bacteria by utilizing the cationic AuNPs attached to poly (para-phenyleneethynylene) (PPE) provided more rapid and efficient identification than conventional plating and culturing. PPE did not fluoresce in the bound state, whereas the presence of bacteria allowed electrostatic interactions between the bacterial surface and the AuNPs that led to the liberation of PPE from the bound conjugate. Free PPE (fluorescent signals) concentrations can be estimated for the rapid quantification of bacteria [139].

### Silver Nanoparticles (Ag NPs)

Application of various antimicrobial substances like metals provides a promising way to control undesirable growth of microorganisms. Basically, the heavy metals have been considered for providing broad-spectrum biocide effects. Among all the metals, the ionic silver considered to have largest antimicrobial activity with long-term biocide properties and low volatility with low toxicity to eukaryotic cells. Furthermore, in recent years, silver has gained popularity because of the spread of antibiotic-resistant Staphylococcus aureus strains, being resistance to silver considered sporadic with a low clinical incidence [140]. The release of silver ions helps to reduce microbial load with sustainable development of various aseptic food containers and antimicrobial surfaces, providing active packaging food systems with promising quality. Very low amount of silver ions (10–100 mg Ag t/kg) is required to achieve biocidal effects using in water or low buffered systems. Interestingly, the antimicrobial activity of silver decreases rapidly in the presence of proteins in food system, and hence, the silver amount required was 50–100 mg Agt kg^−1^ in realistic food applications [141]. For microbial inhibition activity, using silver ions remains inconsistent in complex food matrix without proper standardization or determination of minimum inhibitory concentration (MIC) value which leads to wrong claims. Also, the overuse of silver as nanoparticles leads to molecular basis of resistance in microorganism, which should be documented properly and considered in technological applications [142].

## Toxicological Aspects of Nanomaterials in Food

The field of nanotechnology is growing, and along with it the public concern regarding the toxicity and environmental impact of nanomaterials is also increasing. Nanoparticle-mediated toxicity is stimulated by dynamic, kinetic, and catalytic properties and by functionalization, net particle reactivity, agglomeration, and functional environment [121, 122]. Nanoparticles on the surface of the packaging material are not harmful to human beings, but their translocation and integration into food may affect the human health. The entrance/route, absorption, and distribution of NP in the human body with special attention to their cytotoxicity and genotoxicity were discussed previously [143]. Nanoparticles reach the animal system via skin penetration, ingestion, inhalation, intravenous injections or by the implanted medical apparatus; inside the cells, they interact with the biological macromolecules. Toxicokinetic issues caused by the NP are mainly due to their persistent, non-dissolvable, and nondegradable nature [144]. The lack of consumer awareness, government guidelines, policies, and detection methods for nanotechnology risk assessment warrants better understanding of nanomaterial-based toxicity characterization and regulatory processes. The toxicity increases as the size of the metal-based NP decreases [145]. Nanoparticles are highly reactive substances that can readily cross the membrane barriers and capillaries resulting in different toxicokinetic and toxicodynamic properties. Some NPs bind to proteins and enzymes and result in the stimulation of ROS production and oxidative stress. ROS accumulation causes degeneration of mitochondria and induces apoptosis [146]. Most of the NP-imposed toxicity studies carried out in animals revealed that NP could induce strong toxicity to various organs (liver, kidneys) and immune system. Lack of studies on the impact of NP on human health warrants extensive studies to be carried out. Although silver NP has been used in many commercial nanoproducts, only a few in vivo toxicological studies with silver NP have been conducted using the mammalian models (e.g., mice and rats) [147]. Tumor-like changes have been observed in the human cells exposed to TiO_2_ NP. Different cell lines of the lung, GI tract, and skin were used for the evaluation of nanomaterial-induced toxicity in in vitro conditions [148, 149].

International Conference on Harmonization (ICH) and Organization for Economic Co-operation and Development (OECD) proposed the widely accepted genotoxicity detection methods. These methods were used to determine gene mutations, DNA breaks, Ames test (*Salmonella*-based mutagenicity assay), and mammalian cell assays including mouse lymphoma gene mutation assay (MLA), comet assay for apoptotic induction, micronucleus (MN) estimation, and in vivo animal experiments [150, 151]. Modifications were made in the comet assay by the utilization of bacterial enzymes for the detection of oxidized DNA bases and the quantification of oxidative DNA damage. The significant negative results obtained in the in vivo comet and MN assay as compared to in vitro comet assay are mainly due to the potential DNA repairing ability of animal models. However, Ames test and the chromosomal aberration tests are not reliable for the detection of nanoproduct related toxicity assessments [152, 153]. The in vitro cellular mutagenesis system such as the investigation of epithelial cells from the lungs of the treated animals utilizing bronchoalveolar lavage fluid (BAL), neutrophil level in bronchoalveolar lavage fluid from chronic inflammation, and the correlation between bronchoalveolar lavage fluid-based neutrophil content and the extent of DNA strand damage is determined by in vivo comet assay using lung epithelial cells of the treated animals and hypoxanthin–phosphoribosyltransferase (HPRT) assay. Moreover, in vivo micronucleus and comet assays are considered suitable for the decision-making process by the regulatory authorities [150, 154].

The uptake of exogenous materials, including nanomaterials, induced genetic damages in the cells and animal systems resulting in genotoxicity, which is grouped into primary and secondary genotoxicity. The direct contact between the NP and the genomic DNA without induction of inflammatory reactions is known as primary genotoxicity. This has been reported for some specific nanosilver materials, particulate material of asbestos, and crystalline silica [150, 151]. The indirect method of primary genotoxicity is via the generation of ROS in the NP-induced target cells or the reduction in intracellular antioxidants. Quartz particles induced primary genotoxicity through ROS generation from mitochondria that causes damage to DNA, TiO_2_ and C_60_ fullerenes induced genotoxicity via the formation of peroxynitrite and ZnO NP altered the level of hydroperoxide ions, ROS, malondialdehyde (MDA) concentration, and lactate dehydrogenase activity (LDH) which resulted in DNA fragmentation [155, 156]. The disruption of membrane integrity, suppression of DNA repair processes, and decreased ATP levels leading to alternative repairing processes in the nucleus are also reported for primary genotoxicity [157]. In secondary genotoxicity, macrophages and neutrophils are activated by the nanomaterials that cause inflammatory reactions along with genetic damage. The ROS and reactive nitrogen species (RNS) and the mediators of phagocytes are responsible for the inflammation-associated DNA damages [151]. The ZnO NP-induced oxidative and nitrative stresses cause elevated inflammatory reactions and genotoxicity in the human monocyte cells. The long-term application of nanoscale granular bio-persistent particles causes chronic inflammation as well as secondary genotoxicity [158]. The physical and chemical parameters potentially affecting ROS generation and genotoxicity induction include particle size, surface, shape, charges, particle dissolution, the ions from nanometals and metal oxides, UV-mediated induction, aggregation, route of interaction with cells, inflammation, and pH of the medium [159]. Prolonged oxidative stress arising from extreme generation of ROS and obstruction in the regular physiological redox-regulated functions causes detrimental toxic effects at the cellular level which results in DNA damage, uncontrolled cell signaling, altered cell motility, cytotoxicity, apoptosis, and tumor formation [160]. The frequent exposure to NP affects various organs including inflammatory, immune and cardiovascular systems [161].

DNA fragmentation results from DNA single-strand breaks, double-strand breaks, oxidative damage, or chromosomal damage. The tumor suppressor gene *p53* is involved in the cellular repairing processes via the initiation of cell cycle arrest, DNA repair, and senescence. Apoptosis and phagocytosis are involved in the degradation of severely damaged cells. The cells not cleared by apoptosis or phagocytosis result in the formation of cancerous cells. Interestingly, microarray and global gene expression-mediated signaling investigations indicated that silver NP-induced genotoxicity through ROS generation, DNA damage, chromosome instability, mitosis suppression, and immune response activation occurs through the JAK-STAT signal transduction pathway [162]. The double-strand breaks, and cell cycle arrest initiated by TiO_2_ NP stimulated the expression of ataxia telangiectasia-mutated kinase (ATM), *p53*, *CdC*-*2* followed by the suppression of *H2AX*, ATM-and-Rad3-related (*ATR*), *cyclin B1* which proved the genotoxic characteristics of TiO_2_ NP [163].

ZnO NP possesses various unique characteristics like semiconductor property, biocompatibility, pyroelectric, and piezoelectric properties. Due to their antimicrobial nature, they are used in the food industry for food packaging, smart packaging as well as in the nutritional additives. ZnO NP is less toxic compared to other nanomaterials used in the food industry. However, the potential chromosomal damage, single- and double-strand DNA damages were found in the alloy form of Cu–Zn nanoparticles (ANPs) [164, 165]. In plant systems, lower concentrations (10 nM) of 3-mercaptopropanoic acid–CdSe/ZnS quantum dots induced cytotoxicity and genotoxicity. The NP uptake induces the generation of oxidative stresses (ROS, RNS) and lipid peroxidation in the biological systems that play a crucial role in DNA damage, membrane disintegration, and cell death. The bioavailability, fate, behavior, disposition, and toxicity of NP in the environment should be studied in detail to eradicate the problems associated with nanotechnology in food industry [166].

## Safety Concerns and Regulatory Laws

Nanotechnology application in the food industry is tremendous, beginning with ingredients to packaging as well as in the analysis of food products. Apart from their potential uses, their interaction with food system raises a concern about human and animal health. The nanoformulated products are toxic to plants and animals, and no standard regulatory laws regarding their use in food and agri-sector have been introduced so far. Therefore, effective guidelines and policies are required for the safer utilization of nanoparticles in food industry. The regulatory body USFDA is involved in the regulation of nanofoods and food packaging in the USA. Food Standards Australia and New Zealand (FSANZ), a regulatory body under the Food Standards Code actively participates in the regulation of nanofood additives and ingredients in Australia [167, 168]. Risk assessment of nanotechnology in the European Union is performed by the Scientific Committee on Emerging and Newly Identified Health Risks (SCENIHR). Regulations of the European Union emphasized that the nanotechnology-based food ingredients should undergo safety assessment before being authorized for human use [169]. The nanofood or food ingredients are completely covered by the European Union Novel Foods Regulation (EC 258-97). The re-evaluation program by European Food Safety Authority (EFSA) suggested that the authorized nanoadditives before 2009 and food packaging materials should be treated as per the re-evaluation program. While Japan and China are the major nanomaterial producing countries, they do not have proper nanotechnology-specific regulations [170]. The lack of food regulations in several countries is due to less information regarding exposure, availability, and toxicity to human. Due to the emerging regulatory problems, several countries have demanded a regulatory system for handling risks associated with the nanofood. Complete government guidelines and legislations, as well as rigorous toxicological screening methodologies are essential for the legal nanotechnological applications. A widely accepted international regulatory system is urgently required for the regulation of the utilization of nanoparticles in food industry [171, 172].

## Conclusion and Future Perspective

Nanotechnology plays a major role in the food sector through the quality food production ends with advanced processing, packaging, and long-term storage, provided enormous growth in food industry through enhancement in food quality by improving its flavor and texture. The nanomaterials and nanosensors help the consumers providing information on the state of the food inside and its nutritional status with enhanced security through pathogen detection. Most of the food bioactives against various diseases are hydrophobic in nature having least bioavailability and stability; thus, the nanotechnology-based delivery systems provided an enhanced bioavailability and targeted delivery of food bioactive compounds. The nanotechnology-based foods give significant challenges to both government and industry, ensuring the consumer confidence and acceptance for nanofoods available in market. Active utilization of nanocolloidal particles in different branches of food industry, such as food quality, safety, nutrition, processing, and packaging, has been widely reported recently. The properties and behavior of colloidal particles are important to design foods which are safer and healthier with improved quality and sustainability.

Nanoparticles are manufactured all over the world, though very few countries possess the standard regulatory rules for the utilization of nanotechnology in food products. Insufficient scientific exploration on nanosystems creates difficulties in arriving at any conclusions regarding their efficacy. The applications of nanoparticles in food packaging are less harmful than the utilization of nanoparticles as a food ingredient. There is always a threat that nanomaterials may enter the food chain through the air, water, and soil during their manufacture and usage leading to DNA damage, cell membrane disruption, and cell death. So far, very few in vivo studies have been conducted on the effects of nanofoods in human and animal health. There should be appropriate labeling and regulations advised for marketing of nanofoods which can help to increase consumer acceptability. Thus, utilization of these nanotechnologies, if managed and regulated correctly, can play a significant role in improving food processing and product quality which will benefited for human health and well-being.
